# Cognitive Stimulation in Older Adults with Dementia: A Systematic Review

**DOI:** 10.3390/jcm14207225

**Published:** 2025-10-13

**Authors:** María Jiménez-Palomares, Olga Montero-Barrero, Elisa María Garrido-Ardila, Alicia Gibello-Rufo, Blanca González-Sánchez, Juan Rodríguez-Mansilla

**Affiliations:** 1 ADOLOR Research Group, Department of Medical-Surgical Therapy, Medicine Faculty and Health Sciences, University of Extremadura, 06006 Badajoz, Spain; mariajp@unex.es (M.J.-P.); egarridoa@unex.es (E.M.G.-A.); jrodman@unex.es (J.R.-M.); 2 San Miguel Nursing Home, 06411 Santa Amalia, Spain; olgamnbrto@gmail.com; 3 Faculty of Nursing and Occupational Therapy, University of Extremadura, 06006 Badajoz, Spain; aliciagr@unex.es

**Keywords:** cognitive stimulation, dementia and occupational therapy, non-pharmacological therapies in dementia

## Abstract

**Background**: Dementia is a condition that affects the components of cognitive functions that are responsible for processing thought. There is no cure, but both pharmacological and non-pharmacological treatment helps to slow its progression. Presently, there is an increasing interest in non-pharmacological treatment, including cognitive stimulation, which aims to improve the person’s preserved abilities in order to slow down the progression of the disease while maintaining the current state for as long as possible. The aim of this systematic review is to analyse the effects of cognitive stimulation in older people with dementia. **Methods**: This systematic review was conducted in the Pubmed, OTSeeker, ScienceDirect, Dialnet, and Scopus databases. The inclusion criteria were controlled trials, randomised and non-randomised clinical trials, and pilot studies that applied cognitive stimulation to older people with dementia or compared this therapy with another type of non-pharmacological intervention. **Results**: Twenty-one studies were included in the review. Most of the articles showed that the intervention group achieved better cognitive performance than the control group after completing the cognitive stimulation intervention. Four of the studies assessed caregivers and, in two of the them, improvements in the caregiver’s relationship with the person with dementia were achieved and the caregivers also improved their health-related quality of life. **Conclusions**: According to the results, cognitive stimulation does influence older adults with dementia, especially on cognitive functions. The results also indicate that cognitive stimulation can be beneficial for the caregivers because this therapy has positive effects on their quality of life as related to both their health and their relationship with the person with the disease. However, more research is needed, especially regarding the quality of life of patients with the disease.

## 1. Introduction

As described by the World Health Organisation (WHO) [1], dementia is a syndrome that affects memory, orientation, calculation, comprehension, and language, among other components of cognitive functions which are responsible for processing thought. This is often accompanied by other impairments such as emotional control, social behaviour, or motivation [2].

It is one of the most common chronic diseases; the prevalence in Spain in people over 65 years of age is 4–9% and in people over 90 years of age it can reach 31–54%, and it is usually more common in women. There are no official figures for the total number of cases in Spain, but the estimate is between 500,000 and 600,000 cases.

Prevalence studies conducted worldwide show a consistent trend: the risk of dementia increases with age. Currently, it is estimated that approximately 46 million people live with this condition.

If the current rate of population ageing continues and the incidence rates do not change significantly, it is estimated that the number of people with dementia could reach 131 million by 2050 [2].

Dementia can present itself in multiple forms, which can often coexist with each other. The most common from is Alzheimer’s disease, but there are other forms of dementia such as frontotemporal dementia, Lewy body dementia, or vascular dementia [3].

For decades, drug treatments have been the only option for controlling the symptoms of the disease. However, advances in research have generated a paradigm shift in the form of biologic drugs that work by reducing or eliminating the accumulation of β-amyloid, particularly in cases of Alzheimer’s disease [4]. These treatments can significantly change the course of the disease for people in the early stages, although they are also associated with adverse events [5]. Treatments that delay the progression of the disease and its symptoms include non-pharmacological therapies such as cognitive stimulation and cognitive therapy [3].

The main objective of this therapy is to improve the person’s preserved abilities in order to slow the development of the disease while maintaining the person’s current state for as long as possible. It is based on the principles of brain plasticity and how the brain is able to adapt and form new connections [6]. Research on the benefits of non-pharmacological therapies, such as cognitive stimulation, in people with dementia has been the subject of study for years [7,8]. Despite the advances in the field, it is still important to continue developing research in this area and to establish formal, standardised protocols for occupational assessment and intervention processes.

The available literature indicates that there is a shortage of well-structured clinical trials, which represents a significant methodological limitation. This shortcoming means that the available results must be interpreted with caution, as they do not allow for definitive conclusions to be drawn [8].

Therefore, the aim of this review was to analyse the effects of cognitive stimulation in older adults with dementia.

## 2. Materials and Methods

### 2.1. Study Design

This study is a systematic review that followed the PRISMA guidelines and included controlled trials, randomised and non-randomised clinical trials, and pilot studies [9]. The protocol for this systematic review was not registered with PRISMA.

Articles published in English and Spanish from 2012 to 2025 that conducted cognitive stimulation treatments with older adults with dementia were included in this systematic review.

### 2.2. Eligibility Criteria

Eligibility criteria were defined following the PICOS framework (Population, Intervention, Comparison, Outcome, Study design).

Inclusion criteria (PICOS framework):

- Population: Older adults (male and female) with diagnosis of major neurocognitive disorder due to dementia who live in the community and/or nursing homes.
- Interventions: Cognitive stimulation for older adults with dementia.
- Comparison with another type of non-pharmacological intervention or with a control group.
- Outcome measures: Cognitive benefits, quality of life of the patient and caregiver, executive functions, and the relationship between the user and their caregiver.
- Study design: Controlled trials, randomised clinical trials, non-randomised clinical trials, and pilot studies.
- Studies published from 2012 to 2025.
- Publications in English or Spanish.

Exclusion criteria

All studies using cognitive stimulation in combination with another intervention and not as the sole experimental treatment. This is because the use of another treatment alongside the experimental intervention (cognitive stimulation) may bias the results of the latter.

### 2.3. Search Strategy

The search was carried out in the electronic databases Pubmed, OTSeeker, ScienceDirect, Dialnet, and Scopus.

Three search strategies were carried out in August 2025 using the following keywords ‘cognitive stimulation’, ‘dementia’, and ‘occupational therapy’.

The search process is reflected in the Table 1:

### 2.4. Study Selection

Based on the results obtained via the different search engines mentioned above, two independent researchers selected the articles on the basis of whether or not they met the inclusion criteria. In cases of disagreement, a third researcher intervened to make the final decision. Firstly, articles were selected on the basis of their titles when searching in the different databases. Next, the abstracts were read to determine whether the selected studies were eligible for this review. The full-text studies were then read and analysed in detail. Finally, those studies that met the inclusion criteria were included in the review.

### 2.5. Methodological Quality Analysis

Methodological quality was assessed using the PEDro scale. This scale was developed to assess the methodological quality of randomised clinical trials. It is an 11-item tool (scored yes/no) yielding a 0–10 score (Item 1 is not scored as it assesses external validity); higher scores indicate better quality, categorised as excellent (9–10), good (6–8), fair (4–5), or poor (≤3). Items 2–9 evaluate internal validity (randomization, blinding, and attrition), while Items 10–11 assess statistical reporting adequacy [10]. A trained reviewer conducted all quality assessments to ensure consistency in application of the scoring criteria.

## 3. Results

**Figure 1:**
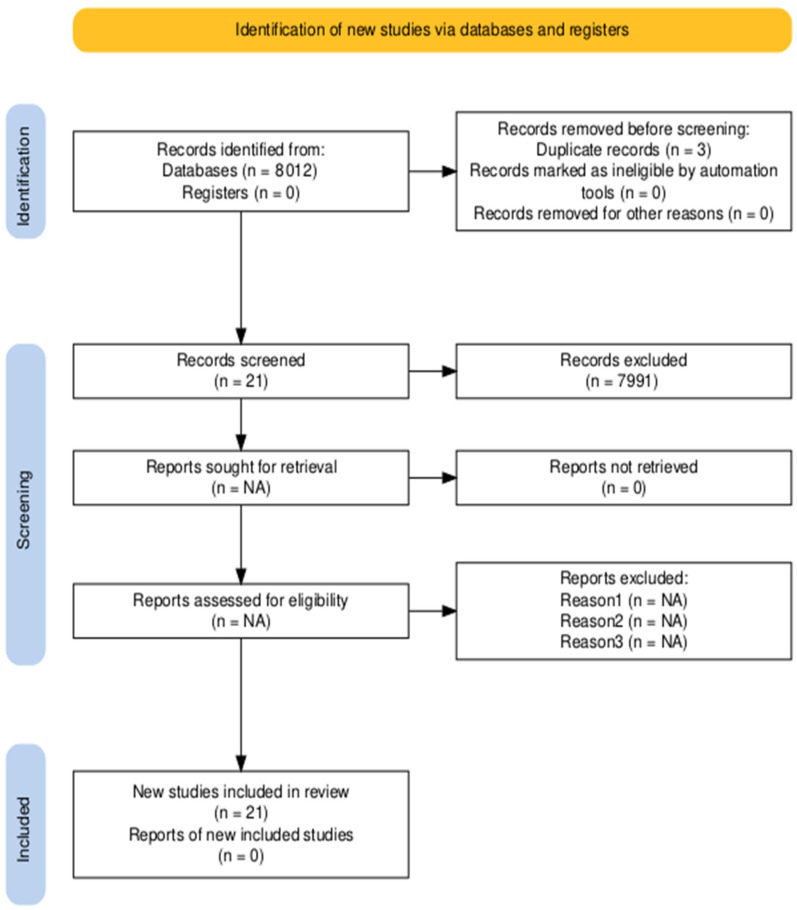
PRISMA flow chart.

Of the 8012 potentially relevant articles, 21 were included in this review, according to the inclusion criteria outlined above. The study selection process is shown in the PRISMA flow chart (Figure 1).

Key characteristics of the included studies are summarised in Table 2.

Of the 21 studies included in this review, we found that 12 are randomised controlled trials [11,12,13,14,15,16,17,18,19,20,21,22] in which the benefits of cognitive stimulation were compared with other treatments such as social activities [11], musical activities, creative activities, and sports [22], with a brief cognitive stimulation programme [11] or with a combination of cognitive sessions and the participant’s usual treatment [21]. Another large group of randomised controlled trials compared cognitive stimulation with the usual treatment received by participants [13,14,15,16,17,18,19,20]. In addition, this review includes two pilot randomised controlled trials with a usual treatment control group [23,24].

We identified two multicentre randomised controlled trials [25,26]; however, only one of them compared cognitive stimulation across different types of dementia, specifically vascular dementia and Alzheimer’s disease [26].

In contrast, we found four quasi-experimental pretest–posttest studies [27,28,29,30], of which only one was multicentre [31].

Among the studies that specified the type of dementia, Alzheimer’s disease was the most prevalent [16,18,20,21,22,25]. All studies investigated the effects of cognitive stimulation in cases of mild or moderate dementia.

Focusing on the type of cognitive stimulation programmes applied in the studies included in this review, we found that most of the studies were based on the work of Spector et al. [13,14,16,19,21,22,24,26,28,29,31]. This programme consists of 14 sessions, each lasting 45 min, delivered twice a week over a period of seven weeks. The sessions include physical games, sounds, childhood memories, food, current events, faces/scenes, word association, creativity, object categorisation, orientation, money use, number games, word games, and team competitions.

One study used the game CoS-Play [11], based on the Play Intervention for Dementia developed in 2013. Justo-Henriques et al. [23] employed the cognitive stimulation tools Bingos Seniores^®^, which included activities such as the “Journey to the Past” bingo (inspired by reminiscence therapy and aimed at stimulating episodic memory), the “Fruit Bingo” (which enhances short-term and semantic memory), and the “Sound Bingo” (which targets sensory memory, semantic processing, and hand–eye coordination). Additionally, Roletas da Memória^®^ offers exercises in mathematics, the Portuguese language, and daily living skills [23].

Another study applied the “Making a Difference” programme [30], which has been validated and adapted for the Portuguese population. The remaining studies included in this review conducted specific cognitive stimulation programmes [12,15,17,18,20,25,27].

**Table 2:** Characteristics of the studies.

Authors	Objective	Intervention	Duration	Outcome Measures	Assessment Tools	**Results**
Cheung et al. [11]	To study the feasibility and preliminary efficacy of a cognitive stimulation games intervention on cognitive functions.	N = 30 Intervention group (n = 15): cognitive stimulation through CoS-Play. Control group (n = 15): social activities that followed a similar pattern to the intervention group.	8 weekly sessions of 45 to 60 min.	Cognitive functions. Verbal fluency.	MoCA FOME FVFT Questions to centre staff	The results showed significant differences from the intervention group to the control group with respect to the functions of memory storage and retrieval, with a mean of 5.92 in the intervention group and 4.12 in the control group. In terms of global cognition and verbal fluency no differences were found between the groups. Acceptance and integration was good, but regarding practicality they considered that a lot of manpower was needed.
Alves et al. [12]	To develop a relevant intervention, enhance cognitive function, foster social engagement, and improve participants’ quality of life.	N = 20 Intervention group (n = 10): full cognitive stimulation programme. Waiting list group (n = 10): cognitive stimulation but shorter.	7 sessions of 1 h each, during 1 month and a half. Each session had 2 levels of difficulty.	Cognitive functioning. Social interaction and engagement. Quality of life.	MMSE ADAS IADL Nonpharmacologic all Therapy Experience Scale.	The results show that the waiting list group showed higher global cognitive functioning than the intervention group (*p* = 0.03). For the other variables, no significant differences were found between the two groups.
Coen et al. [13]	To evaluate the efficacy of cognitive stimulation therapy, replicating the methods of Spector et al. in the *British Journal of Psychiatry*.	N = 27 Intervention group (n = 14): cognitive stimulation in conjunction with usual therapies. Control group (n = 13): continued to receive usual therapies.	14 sessions of 45 min, twice a week for seven weeks.	Cognitive performance. Quality of life.	MMSE WHOQOL	The intervention group improved cognitive performance compared to the control group (*p* = 0.013). Regarding quality of life, no significant differences were found between the groups (*p* = 0.055).
Katsuo Yamanaka et al. [14]	To develop and examine whether the Japanese version of group cognitive stimulation therapy produces improvement in cognitive function and quality of life in people with mild to moderate dementia.	N = 56 Intervention group (n = 26): Japanese version of group cognitive stimulation therapy. Control group (n = 30): usual activities.	14 sessions, twice a week for 7 weeks.	Cognition. Quality of life. Mood.	COGNISTAT. MMSEEQ-5D QoL-AD Facial mood scale	The results showed significant improvements in cognitive functions and mood in the intervention group compared to the control group (*p* < 0.01). Quality of life improved in the intervention group when rated by caregivers, although there was no improvement when rated by the participants themselves.
Kolanowski et al. [15].	To analyse whether cognitive stimulation activities reduce the duration and severity of delirium and improve cognitive and physical function to a greater extent than usual care.	N = 283 Intervention group (n = 141): cognitive stimulation. Control group (n = 142): usual treatment.	30 days	Duration and severity of delirium. Cognitive function. Physical function.	CAM DRS BI DF, MOC CLOX	The results of the study showed that the severity and duration of delirium were similar in both groups (*p* = 0.37). While improvements in constructive praxis (*p* = 0.0003) and executive function (*p* = 0.03) were seen in the intervention group compared to the control group. The length of stay was also shorter in the intervention group than in the control group (*p* = 0.01).
Aguirre et al. [16]	To study the effect of cognitive stimulation therapy on the general health status of caregivers of people with dementia living in the community who attend the intervention.	N = 85 Intervention group (n = 41): users with dementia included here received cognitive stimulation. Control group (n = 44): users continued with usual care.	7 weeks, 14 sessions of standard cognitive stimulation therapy. Subsequently, 24 weeks of maintenance cognitive stimulation therapy	General well being state	EQ-5D (for carers). SF-12 (for self-assessment of users).	The results showed that there is no evidence between the two groups that cognitive stimulation produces improvements in the caregivers of family members with dementia attending the intervention.
Vasiliki Orgeta et al. [25]	To assess the clinical effectiveness and cost-effectiveness of individual caregiver-led cognitive stimulation therapy for people with dementia and their family caregivers, compared to treatment as usual.	N = 356 Individuals were randomly assigned to each group. Intervention group (n = 180): cognitive stimulation at home led by their caregiver. Control group (n = 176): treatment as usual led by their caregiver.	3 sessions of 30 min (each) per week and for 25 weeks	Cognition. Quality of life and relationships. Behavioural, psychological and depressive symptoms. Activities of daily living.	SF-12 EQ-5D. ADAS-Cog. QoL-AD	The intervention group showed improved relationships with caregivers (*p* = 0.02), who also reported better health-related quality of life (*p* = 0.01) and communication. Qualitative data suggested fewer depressive symptoms among caregivers attending more sessions. No significant differences were found for other variables. The therapy also led to greater health gains and cost savings.
Martin Orrell et al. [17]	To evaluate the effectiveness of a caregiver-led, home-based individual cognitive stimulation therapy programme in improving cognition and quality of life of the person with dementia and the mental and physical health (well-being) of the caregiver.	N = 356 Intervention group (n = 180): they received cognitive stimulation led by their usual caregiver. Control group (n = 176): they continued with the usual therapies but directed by their caregiver.	3 sessions of 30 min per week for 25 weeks (75 sessions in total).	Cognition. Self-reported quality of life. General health status of the caregiver. Quality of the relationship with the caregiver.	ADAS-Cog. QoL-AD SF-12 EQ-5D	The results showed that people with dementia in the intervention group improved the quality of their relationship with their caregiver (*p* = 0.02) and also the caregivers in this group improved their health-related quality of life (*p* = 0.01) compared to the control group. In the rest of the variables there were no significant differences between the two groups.
DeokJu Kim [18]	To determine the effectiveness of reconverted occupational therapy programmes.	N = 35 Experimental group (n = 18): received the reminiscence-based occupational therapy programme. Control group (n = 17): continued to receive the regular activities offered by their day centres.	24 sessions, 5 times a week and for 1 h per session.	Cognitive functions. Depression. Quality of life. Activities of daily living.	FIM. K-MMSE. SMCQ GDS-SF-K GQOL-D.	The results showed that the experimental group had an improvement in cognitive functions (*p* < 0.05), a reduction in depression (*p* < 0.05) and a higher quality of life (*p* < 0.01) compared to the control group.
Justo- Henriques et al. [23]	To assess the efficacy, feasibility and acceptability of a long-term individual cognitive stimulation intervention for people with mild neurocognitive impairment.	N = 30 Intervention group (n = 15): cognitive stimulation. Control group (n = 15): 15 routine interventions.	88 individual sessions of 45 min each, twice a week.	Cognitive performance. Depressive symptoms. Level of autonomy in activities of daily living.	MMSE. MoCA GDSBI. Questionnaire of sociodemographic characterisation. Registration sheet.	The results of the study showed that the intervention group obtained a significant improvement in global cognitive performance (d = 0.83) in particular in the area of language and less depressive symptomatology (d = 0.93) compared to the control group. No differences were found between the two groups in the autonomy in performing activities of daily living (*p* = 0.34). Only 6.7% of the participants dropped out of the study and of the intervention group the participants attended an average of 83 ± 12.1 sessions.
Cintoli et al. [27]	Evaluate the feasibility and effectiveness of a cognitive stimulation intervention for dementia patients, contributing to the development of more accessible and personalised therapeutic strategies	N = 19 dementia patients, with 12 participating in in-person treatment and 7 engaged remotely. Cognitive stimulation protocol: memory, attention, and problem-solving exercises, guided by a clinical psychologist. The protocol was adapted to an online format, maintaining the characteristics of the in-person implementation.	Eight weekly, 1 h individual sessions	Cognitive performance. Level of autonomy in activities of daily living. Satisfaction for both patients and caregivers.	MMSE ADL Scale, IADL Scale VAS Likert scale	No differences were found at the beginning or end of the study inoverall cognitive functioning or in the ability to performactivities of daily living (ADL) and instrumental activities of daily living (IADL) in either group. The Cognitive stimulation delivered both in-person and via videoconferencing, is feasible and well-received by both patients and caregivers.
Pérez-Sáez et al. [28]	Adaptation of the cognitive stimulation therapy (CST) from the United Kingdom to the Spanish cultural context, implementing sessions.	N = 6 Intervention adapted from the cognitive stimulation therapy Spain (CST-ES) and maintenance cognitive stimulation therapy MC Spain (ST-ES) CST programme consists of 14 45 min sessions that are conducted twice a week for 7 weeks MCST program that includes 24 additional sessions is delivered after the 7 weeks of CST, at a rate of one session per week.	14 45 min sessions that are conducted twice a week for 7 weeks	Cognitive performance. Level of autonomy in activities of daily living. Depressive symptoms. Quality of life.	MMSE, CAMCOG-R, GDS-15, QoL-AD BI IADL	The results are similar to those of other cultural adaptations, showing positive changes to general cognition and quality of life following the implementation of CST. However, no improvements were observed in activities of daily living. After the MCST-ES programme, cognitive scores remained stable, and there was a non-significant decline in quality of life.
Spector et al. [19]	Assess the feasibility and acceptability of online cognitive stimulation therapy (CST) or “virtual” cognitive stimulation therapy (vCST).	N = 46 Intervention group (n = 24) vCST: involved 14 60 min group sessions delivered twice a week for 7 weeks. Control group treatment as usual (TAU) (n = 22).	14 60-min group sessions delivered twice a week for 7 weeks.	Cognition. Quality of life. Depression.	ADAS-Cog GDS-15 MOCA BLIND QoL-AD	No statistically significant differences were found between the vCST and treatment as usual groups in any of the outcome measures. Although at a qualitative level, there were reports of improvements in the outcomes.
Piras et al. [26]	To compare the effectiveness of the Italian Cognitive Stimulation Therapy (CST-IT), applied in a previous multicenter controlled clinical trial, across two distinct cohorts of individuals with Alzheimer’s disease and vascular dementia in the mild-to-moderate stage	The Italian adaptation of the original CST protocol developed by Spector and colleagues N = 58 Alzheimer disease Group n = 30 Vascular Dementia Group n = 27	20 sessions over a period of 23 weeks.	General cognitive functioning Communicative abilities Mood Behaviour Perceived quality of life	MMSE ADAS- Cog NLT CSDD NPI QoL-AD	CST-IT achieved clinically significant improvements to general cognition and communicative abilities. Depressive symptoms decreased more notably in patients with Alzheimer’s disease, while quality of life showed a slight improvement in those with vascular dementia. Improvements in narrative abilities were observed in patients with vascular dementia. Post-intervention gains in depressive symptoms persisted in Alzheimer’s disease but not in vascular dementia, although the benefits in quality of life remained stable in the latter group.
Bertrand et al. [24]	Explore the impact of a Brazilian adapted version of Cognitive Stimulation Therapy (CST-Brasil) on the level of awareness.t	N = 47 Experimental Group: cognitive stimulation therapy n = 24. Control Group: treatment as usual (TAU) N = 23.	7 weeks	Metacognitive: level of awareness.	ASPIDD	Awareness of the disease increased in both groups. However, only those who received Cognitive Stimulation Therapy showed improvements in awareness of their cognitive abilities
Dudley et al. [29]	Adaptation of the cognitive stimulation therapy(CST) from the United Kingdom to the Māori cultural context, implementing sessions	N = 15 Two CST programmes: the 14 45 min sessions that are conducted twice a week for 7 weeks.	7 weeks	Cognition. Quality of life.	RUDAS WHOQOL-BREF	There was a statistically significant improvement in cognition (RUDAS: pre = 17.7, post = 19.4, *p* = 0.003) and in the WHOQOL subscales of physical (pre = 75.9, post = 88.5, *p* = 0.003), psychological (pre = 72.7, post = 81.3, *p* = 0.024) and environment (pre 80.6, post = 88.0, *p* = 0.006).
Cunha [30]	To evaluate the effectiveness of individual cognitive stimulation interventions	N = 21 Three sessions per week for a total of 36 sessions the “Making a Difference 3”. An Individual Cognitive Stimulation Program.	12 weeks	Cognition. Quality of life neuropsychiatric symptoms of older adults with dementia. Quality of the relationship between the older adult with dementia and the caregiver.	GDS-15 QCPR QoL-AD SLUMS NPI–Q	There were statistically significant improvements in neuropsychiatric symptoms (*p* = 0.042) and cognition (*p* = 0.038) after the programme was administered.
Justo-Henriques [20]	To assess the efficacy of a long-term individual cognitive stimulation intervention on people with mild neurocognitive disorder	N = 82 Cognitive stimulation intervention group n = 41 Control group n = 41	88 individual format sessions of approximately 45 min, twice per week.	Cognition. Depressive symptomatology. Autonomy level in activities of daily living.	MMSE MoCA GDS-15 BI	Significant improvement on cognition and depressive symptomatology in the intervention group compared to the control group were found intra-intervention (6 months) and post-intervention (12 months). Younger participants and those with better cognitive status at the beginning of the study achieved better results.
Coşkun and Çuhadar [21]	Evaluate the effects of Cognitive Stimulation Therapy on activities of daily living, depression, and life satisfaction in older adults with dementia in nursing homes.	N = 60 Intervention group n = 30: Cognitive Stimulation Therapy. Control group n = 30: received only two sessions in week 2, and they continued their daily lives and routine treatments.	9 weeks. 14 sessions approximately 45 min	Activities of daily living. Depression. Life satisfaction.	SMMSE BADLIIADLS CSDD SWLS	Statistically significant improvements were observed in the intervention group in BADLI, IADLS, and CSDD, both in the post-test and in the follow-up. While Life satisfaction showed statistically significant improvements only in the post-test.
Atay, E., & Bahadır Yılmaz, E [22].	Determine the effect of Cognitive Stimulation Therapy (CST) on apathy, loneliness, anxiety, and activities of daily living of individuals with Alzheimer’s disease.	N = 52 Intervention group n = 26: Cognitive Stimulation Therapy. Control group n = 26: in routine unstructured music, sports, and art activities at the centre.	14 sessions, twice a week.	Apathy. Loneliness. Anxiety. Activities of daily living.	MMSE AES-C UCLA UCLA-SF GAS DAD	A significant reduction in apathy, loneliness, and anxiety was observed in the intervention group compared to the control group. Following the application of Cognitive Stimulation Therapy, significant differences were observed in the intervention group on the AES-C, UCLA, GAS and DAD
Zubatsky et al. [31]	Explored differences in cognitive function, mood, and quality of life from CST groups both community and residential-based groups.	N = 258 From academic and rural, hospital-based settings in Missouri received s Cognitive Stimulation Therapy (CST).	7 weeks, each session was 1 h. 14 Sessions	Cognitive function. Mood. Quality of life.	SLUMSCSDD QOL-AD	Following the intervention, cognitive function improved in both the community and residential groups. However, participants living in the community showed significant improvements in mood.

NOTE: MoCA: Montreal Cognitive Assessment; FOME: Fuld’s Object Memory Assessment; FVFT: Fuld Verbal Fluency Test; MMSE: Mini-Mental State Examination; ADAS: Alzheimer’s Disease Assessment Scale: IADL: Instrumental Activities of Daily Living; WHOQOL: Quality of Life; COGNISTA: Neurobehavioral Cognitive Status Examination; EQ-5D: European Quality of Life-5; QoL-AD: Quality of Life—Alzheimer’s Disease; CAM: Confusion Assessment Method; DRS: Delirium Rating Scale; BI: Barthel Index; DF: Digits Forward; CLOX: Clock Drawing Executive Test; EQ-5D: EuroQol 5 Dimension; SF-12: 12-Item Short-Form Health Survey; ADAS-Cog: Alzheimer’s Disease Assessment Scale—Cognitive Subscale; FIM: Measurement of Functional Independence; K-MMSE: Korean Mini-Mental State Examination; SMCQ: Subjective Memory Complaints Questionnaire; GDS-SF-K: Geriatric Depression Scale—Short-Form-K; GQOL-D: Geriatric Quality of Life—Dementia; Global Deterioration Scale: GDS; GDS-15, Geriatric Depression Scale—15 items; VAS: Visual Analogue Scale; CAMCOG-R: Cambridge Cognitive Examination—Revised; NLT: Narrative Language Test; CSDD: Cornell Scale for Depression in Dementia; NPI: Neuropsychiatric Inventory; ASPIDD: Assessment Scale of Psychosocial Impact of the Diagnosis of Dementia; RUDAS: Rowland Universal Dementia Assessment Scale; QCPR: Quality of the Carer–Patient Relationship Scale; SLUMS: Saint Louis University Mental Status Test; SMMSE: Standardised Mini-Mental State Examination Test; BADLI: Barthel Activities Of Daily Living Index; CSDD: Cornell Scale For Depression In Dementia; SWLS: Satisfaction with Life Scale; AES-C: Apathy Evaluation Scale—Clinician Version; UCLA: Loneliness Scale—Short Form; GAS: Geriatric Anxiety Scale; DAD: Disability Assessment for Dementia.

### Methodological Quality

The results of the methodological quality assessment can be seen in Table 3.

It should be noted that eight of the studies [11,12,14,15,16,17,19,20,21,22,23,24,25,26] included in this review obtained a score ≥6, indicating that the clinical trials have good internal and external validity, with two of them scoring 9 [15,17,22].

Only two studies analysed obtained a score of 5 [13,18].

## 4. Discussion

The aim of this review was to analyse the effects of cognitive stimulation in older adults with dementia. After applying the search strategy and selection of articles, 21 studies were finally included. The randomised clinical trials analysed showed good internal and external validity and sufficient statistical information to make their results interpretable, according to the Pedro scale with scores ≥6 [11,12,14,15,16,17,19,20,21,22,23,24,25,26].

According to Woods et al. [32], there are still relatively few studies on individual cognitive stimulation. Of the studies included in this review, only five indicate that cognitive intervention was individualised [17,20,23,25,28]. The rest of them either did not specify this aspect [12,15,18,27] or conducted group-based interventions [11,13,14,19,21,22,24,26,29,30,31]. Further research is needed to determine the effectiveness of the different methods of administration.

Out of the studies included in this review, two conducted online cognitive stimulation [19,27]. The first study concluded that online cognitive stimulation is as effective as face-to-face stimulation [27], although both studies only observed maintenance of cognitive functions. There were no statistically significant changes compared with face-to-face cognitive stimulation [27] or with usual treatment [19].

We found that most of the studies included in this review were carried out with participants from treatment centres, such as day centres, nursing homes [11,12,13,14,18,19,22,24,26,28], and outpatient clinics, or were recruited through them [15,20,23,27,29]. One of the included studies compared cognitive stimulation with patients living in the community and those living in nursing homes [31]. The authors concluded that both groups showed improvement in cognition. However, mood improved significantly more in the group of community-dwelling participants.

There is still a limitation regarding who applies the cognitive stimulation treatment. In many studies, this information was not specified [11,15,18,23,25]. In others, it was applied by health professionals, such as psychologists [12,14,16,19,20,21,22,24,26,27,28,29,30,31], occupational therapists [13,18], and by informal caregivers [17,23]. Future studies should address this aspect to determine how this aspect may influence the success of such interventions.

Among the included studies, the most frequent effect obtained after applying cognitive stimulation in people with dementia were improvements in cognitive functions [11,12,13,14,15,18,20,23,26,28,29,30,31] followed by others such as a decrease in depression in people with the disease [18,20,21,23,30,31], a better relationship with their caregivers [17,25] and better health-related quality of life [21,29], and the quality of life of caregivers [17,25,28].

Of the included articles, only one explores metacognition, specifically the level of awareness, anosognosia [24]. The authors of this article attributed positive effects on metacognitive functioning, particularly with regard to awareness of cognitive deficits, to the application of cognitive stimulation. Given the negative impact of a lack of awareness on people with dementia and their carers, these results are relevant in a clinical context. Since improvements in awareness promoted by cognitive stimulation therapy can benefit patients and their families, these findings have clinical implications.

Most of the articles showed that the intervention group achieved better cognitive performance than the control group after completing the cognitive stimulation intervention [16,23] with a large effect size, as in the article by Justo-Henriques et al. [23]. These authors specifically found improvements in the area of language. These results are similar to those of other articles included in the review [11,13,14,18]. However, the effect size found in these articles was smaller than in the study by Kolanowski et al. [15]. It is interesting to highlight that this last study had a sample of 283 participants in comparison with the other articles where the sample was around 30. In this respect, there are authors such as Villalba Agustín et al. [33] who explain how the prefrontal area, where the executive functions and constructive praxes are found, are those most affected by ageing, but that with specific objectives focused on them, they can be improved and new neuronal connections can be formed. Cheung et al. [11], in their study, also assessed the change in cognitive functions when applying cognitive stimulation and these improved in the intervention group in comparison to the control group, although the sample size was 30 participants. Cheung et al. [11] also assessed the verbal fluency of the participants, although it could have not improved because, according to the scientific literature [33], linguistic functions are those that suffer the least deterioration over the years, although stimulation may improve them, but as they are stable, no changes were observed. However, some studies comparing cognitive stimulation in patients with Alzheimer’s-type dementia and vascular dementia did observe improvements in communication skills [26]. Specifically, narrative skills improved in the vascular dementia group. This is the only study that compared the effects of cognitive intervention according to the dementia type.

Despite these positive results, we have also found studies where after the application of cognitive stimulation, there were no changes between the control and intervention groups [16,17,19,25,27]. We consider that this could be because there are times when cognitive stimulation does not improve any function, but it does maintain them [34], as has occurred in the previously mentioned studies that did not show changes, but neither has the intervention group had any detectable decrease or worsening in the outcome measures. In this regard, the review by Woods et al. (2023) also concluded that cognitive stimulation does not produce negative effects [32]. Therefore, it is very important to continue with the stimulation over time because if these functions do not continue to be trained, they will end up being lost and this could lead to a worsening in the quality of life of these people. Focusing on the methodology of the studies that did not obtained improvements, we found that the number of hours and sessions dedicated to cognitive stimulation had been an average of 42 h in total and in 1 month and a half in comparison with the 10 h in total during 3 or 4 months that most of the other studies have obtained differences from the intervention group to the control group [11,13,14,15,17,18,23,25]. This suggests that better improvements can be obtained when the number of sessions is greater in time although the duration of the sessions is shorter. These results coincide with a systematic review conducted in 2023 [32], which indicated that better results are obtained with more frequent sessions. Twice a week or more is more beneficial than once a week [32]. This is supported by McGee and Bratkovich [35] who stated that ‘interventions must be adapted to the speed of processing and the loss of memory and attention that older people have, in this case with dementia, because regular cognitive stimulation over time increases the benefits of this’.

We have only found one study in which the control group obtained improvements in comparison with the intervention group, and that is the study by Alves et al. [12], in which the 17 sessions of cognitive stimulation were applied to the intervention group while the control group continued with the usual treatment. When the intervention group finished, 11 sessions of cognitive stimulation were administered to the control group. The reason that improvements were found in the control group may be due to the fact that they also received stimulation over time, so it is not compared with an inactive control group.

Four of the studies assessed caregivers by applying cognitive stimulation to people with dementia. In two of them [17,25], improvements in the caregiver’s relationship with the person with dementia were obtained and caregivers also improved their health-related quality of life. In one of them, caregivers reported improvements in mood, and if the cognitive stimulation was delivered online, the results showed that it provided more confidence and comfort [27].

Only in one of the studies [17] was communication between the caregiver and patient also improved. In addition, it was found that the more sessions the intervention group carried out, the more the caregivers’ depressive symptomatology decreased compared to those of the control group. On the other hand, the study by Aguirre et al. [16], which also evaluated the caregivers, did not achieve improvements in health-related quality of life in caregivers when applying cognitive stimulation to their users. This may be due, according to the authors, to the fact that they only took into account the variable of health-related quality of life as perceived by the carers themselves and did not contrast it with the view of any person close to the carers to check whether or not this was the case. These authors also believed that greater benefits would be obtained if the cognitive stimulation had been carried out by the caregiver themselves and not by an outsider, as in the two previous studies in which benefits were obtained [17,25].

As the symptoms of dementia increase, the person suffering from dementia loses quality of life due to the symptoms and the reduction in activities that can no longer be carried out independently. In this regard, we found in this review that in two of the studies included, cognitive stimulation did not seem to provide benefits with respect to the quality of life of elderly people with dementia [12,16]. This may be due to the heterogeneity of the groups, the size of the sample, or the degree of dementia suffered by the participants [36]. However, when quality of life was rated by the caregivers, there were improvements from the intervention group to the control group [14], but when it was rated by the caregivers themselves there were no differences. This is due to the subjectivity of the variable since each person perceives it in a different way and, when evaluating it, they express their feelings. Therefore, the differences could be due to the fact that the caregivers may see the patients in a different way from how they see themselves [37].

As dementia and its symptoms progress, people with this condition become increasingly dependent in their daily activities and therefore require a person to give them guidelines or reminders of what to do or how to do it [38]. According to three of the studies included in this review [18,23,28], no differences were evident between the two groups regarding the independence variable. In another of the studies included, there was a maintenance of independence in activities of daily living after the application of a cognitive stimulation programme both in-person and remotely [27].

This may be due to the fact that, according to some authors such as Freund [39], variables with behavioural outcomes are not sensitive enough to detect the impact of cognitive stimulation on them. However, we did find in the scientific literature studies where improvements were obtained for the intervention group in comparison to the control group, when carrying out a cognitive stimulation intervention for elderly people with dementia, with respect to the performance of the participants in activities of daily living [40]. This aspect coincides with one of the studies included in this review [21], in which improvements in basic and instrumental activities were observed not only after the intervention, but also in the follow-up evaluation, in the cognitive stimulation group.

Delirium is almost always associated with dementia and usually increases as the disease progresses, leading to reduced levels of consciousness, sleep disturbance, decreased attention, and inappropriate behaviours [41]. This review has shown that cognitive stimulation can be helpful in improving these aspects of the syndrome [14], specifically both the duration and severity of delirium. In addition to positively influencing neuropsychiatric symptoms such as anxiety, depression [22,30], apathy, and loneliness [22].

Implications for research and clinical practice emerge from this review, as it provides information from several studies on the effects of cognitive stimulation on both people with dementia and their caregivers. However, it should be noted that the search strategy did not follow clearly the defined PICO criteria, which may have led to the loss of some studies. The improvements that this can bring, above all in the cognitive functioning of people suffering from the disease, should continue to be studied with research that has a larger sample size, avoiding biases by carrying out double-blind studies, and with a prolonged follow-up over time with multiple measurement points to check whether the effects of the intervention continue or whether they disappear when the intervention is discontinued.

## 5. Conclusions

According to the results of the studies analysed in this systematic review, cognitive stimulation has a positive effect on older people with dementia, especially on cognitive functions. It has also been shown that this intervention can be beneficial even for the caregivers because it has effects on their quality of life related to both their health and their relationship with the person suffering from the disease. However, more research is needed especially in the area of patients’ quality of life.

## Figures and Tables

**Table 1:** Search strategy.

Key Words	Databases	Total	Excluded	Included
“cognitive stimulation and dementia”	PUBMED	171	158	13
“cognitive stimulation and dementia”	SCIENCEDIRECT	1	1	0
“cognitive stimulation and dementia”	OTSEEKER	35	35	0
“estimulación cognitiva y demencia”	DIALNET	107	107	0
“cognitive stimulation and dementia”	SCOPUS	3877	3.871	6
		<		
“cognitive stimulation and occupational therapy”	PUBMED	34	34	0
“cognitive stimulation and occupational therapy”	SCIENCEDIRECT	1.919	1.918	1
“cognitive stimulation and occupational therapy”	OTSEEEKER	2	1	1
“estimulación cognitiva y terapia ocupacional”	DIALNET	33	33	0
“cognitive stimulation and occupational therapy”	SCOPUS	1.833	1833	0

**Table 3:** Results of the methodological quality assessment with the PEDro scale.

Author	1	2	3	4	5	6	7	8	9	10	11	Total
Aguirre et al. [16] (2014)	1	1	0	1	0	0	0	1	0	1	1	6/11
Cheung et al. [11] (2019)	1	1	1	1	1	0	0	1	0	1	1	8/11
Orgeta et al. [25] (2015)	1	1	1	1	1	0	0	1	0	0	1	7/11
Alves et al. [12] (2014)	1	1	1	0	1	0	1	1	0	1	1	8/11
Orrell et al. [17] (2017)	1	1	1	1	1	0	1	1	0	1	1	9/11
Coen et al. [13] (2011)	1	1	0	0	0	0	0	1	0	1	1	5/11
Yamanaka et al. [14] (2013)	1	1	0	1	0	0	1	1	0	1	1	7/11
Kim [18] (2020)	1	1	0	0	0	0	0	1	0	1	1	5/11
Kolanowski [15] (2016)	1	1	1	1	1	0	1	1	0	1	1	9/11
Justo-Henriques [23] (2019)	1	1	1	1	1	0	0	1	0	1	1	8/11
Spector et al. [19]	1	1	1	0	1	0	1	1	0	1	1	8/11
Piras et al. [26]	1	1	0	1	0	1	1	1	0	1	1	8/11
Bertrand et al. [24]	1	1	0	1	0	1	1	1	0	1	1	8/11
Justo et al. [20]	1	1	0	1	0	0	1	1	0	1	1	7/11
Coşkun et al. [21]	1	1	1	1	1	0	0	1	0	1	1	8/11
Atay. [22]	1	1	1	1	1	0	1	1	0	1	1	9/11

Note: 1: met the criteria; 0: did not meet the criteria.

## Data Availability

Data is available upon reasonable request to the authors.
